# A Single Residue Substitution in the Receptor-Binding Domain of H5N1 Hemagglutinin Is Critical for Packaging into Pseudotyped Lentiviral Particles

**DOI:** 10.1371/journal.pone.0043596

**Published:** 2012-11-02

**Authors:** Dong-Jiang Tang, Yuen-Man Lam, Yu-Lam Siu, Chi-Hong Lam, Shui-Ling Chu, J. S. Malik Peiris, Philippe Buchy, Béatrice Nal, Roberto Bruzzone

**Affiliations:** 1 HKU-Pasteur Research Centre, The University of Hong Kong, Hong Kong, Special Administrative Region, People's Republic of China; 2 Center for Influenza Research – School of Public Health, The University of Hong Kong, Hong Kong, Special Administrative Region, People's Republic of China; 3 Department of Anatomy, The University of Hong Kong, Hong Kong, Special Administrative Region, People's Republic of China; 4 Virology Unit, Institut Pasteur in Cambodia, Phnom Penh, Kingdom of Cambodia; 5 Department of Cell Biology and Infection, Institut Pasteur, Paris, France; German Primate Center, Germany

## Abstract

**Background:**

Serological studies for influenza infection and vaccine response often involve microneutralization and hemagglutination inhibition assays to evaluate neutralizing antibodies against human and avian influenza viruses, including H5N1. We have previously characterized lentiviral particles pseudotyped with H5-HA (H5pp) and validated an H5pp-based assay as a safe alternative for high-throughput serological studies in BSL-2 facilities. Here we show that H5-HAs from different clades do not always give rise to efficient production of H5pp and the underlying mechanisms are addressed.

**Methodology/Findings:**

We have carried out mutational analysis to delineate the molecular determinants responsible for efficient packaging of HA from A/Cambodia/40808/2005 (H5Cam) and A/Anhui/1/2005 (H5Anh) into H5pp. Our results demonstrate that a single A134V mutation in the 130-loop of the receptor binding domain is sufficient to render H5Anh the ability to generate H5Anh-pp efficiently, whereas the reverse V134A mutation greatly hampers production of H5Cam-pp. Although protein expression in total cell lysates is similar for H5Anh and H5Cam, cell surface expression of H5Cam is detected at a significantly higher level than that of H5Anh. We further demonstrate by several independent lines of evidence that the behaviour of H5Anh can be explained by a stronger binding to sialic acid receptors implicating residue 134.

**Conclusions:**

We have identified a single A134V mutation as the molecular determinant in H5-HA for efficient incorporation into H5pp envelope and delineated the underlying mechanism. The reduced binding to sialic acid receptors as a result of the A134V mutation not only exerts a critical influence in pseudotyping efficiency of H5-HA, but has also an impact at the whole virus level. Because A134V substitution has been reported as a naturally occurring mutation in human host, our results may have implications for the understanding of human host adaptation of avian influenza H5N1 viruses.

## Introduction

H5N1 influenza virus is highly pathogenic in poultry, certain bird populations, and has occasionally infected human causing severe clinical outcomes [1]–[3]. Since the first human outbreak in 1997, there have been more than 600 confirmed human cases of H5N1 infection with a mortality rate of approximately 60% [4]. To initiate an infection, like all other subtypes of influenza viruses, H5N1 virus first binds to cell surface glycan receptors via its surface glycoprotein hemagglutinin (HA) and is subsequently internalized via endocytic pathways [5]–[7]. HA is a homotrimeric type I transmembrane glycoprotein, which can be cleaved into HA1 and HA2 subunits [8]. A furin-dependent polybasic cleavage site has been shown to be characteristic of highly pathogenic avian influenza viruses [9], [10], although not all H5-HAs contain the polybasic cleavage site. In cells infected by influenza virus, HA protein is first synthesized as a precursor (HA0), which is then oligomerized, glycosylated and ultimately transported to the plasma membrane where assembly and budding of progeny virions takes place [11]. Then, during the final stage of the virus life cycle, neuraminidase (NA), the second major envelope glycoprotein of influenza, cleaves the terminal sialic acids from the cell surface glycans to allow release of the virus from the host cell [12].

Following the first report of H5N1 outbreak in 1996, the virus has evolved into different clades as shown by the phylogenetic analysis of H5-HA protein sequences [13]–[15]. Currently the spread of H5N1 virus in human population is limited. However, through mutation and reassortment, the virus may become more easily transmissible from bird to human or from human to human, posing a potential pandemic threat to public health worldwide [2], [3]. It is therefore important to fully understand the biology of H5N1 viruses and to develop sensitive and rapid diagnostic methods. However, an obstacle to the study of H5N1 viruses is the stringent safety requirement to work with them. Recently, we and other research groups have developed retroviral particles pseudotyped with H5-HA (H5pp) as an alternative strategy for large scale serological studies [16]–[21]. Similar to the replication-competent virus, H5pp entry requires alpha-2,3 sialic acids, is pH-dependent, and can be neutralized by sera containing anti-H5N1 antibodies [18], thus validating H5pp as very useful and safe tool for a wide range of applications, including entry mechanism studies, sero-diagnosis and drug discovery [16], [18].

In our previous work, we have produced H5pp using the H5-HA of A/Cambodia/40808/2005 (H5Cam), which was isolated from a patient with a lethal infection of H5N1 virus [18]. In the current study, we have analyzed the ability of H5-HAs from different clades of avian influenza virus to pseudotype lentiviral particles and have found that they do not give rise to the same level of efficient H5pp production when compared with H5Cam. In particular, we have carried out a detailed comparison of the expression and cleavage of two H5-HAs, i.e., H5-HA of A/Anhui/1/2005 (H5Anh) and H5Cam, and of their ability to pseudotype lentiviral vector in HEK293T cells. Through several independent lines of evidence we have identified the molecular determinants in H5-HA for efficient incorporation into H5pp envelope and have delineated the underlying mechanism. Our results are discussed in the context of the understanding of human host adaptation of avian influenza H5N1 viruses.

## Results

### The ability of H5-HA to pseudotype lentiviral particles does not correlate with HA protein expression level in producer cells

**Figure 1:**
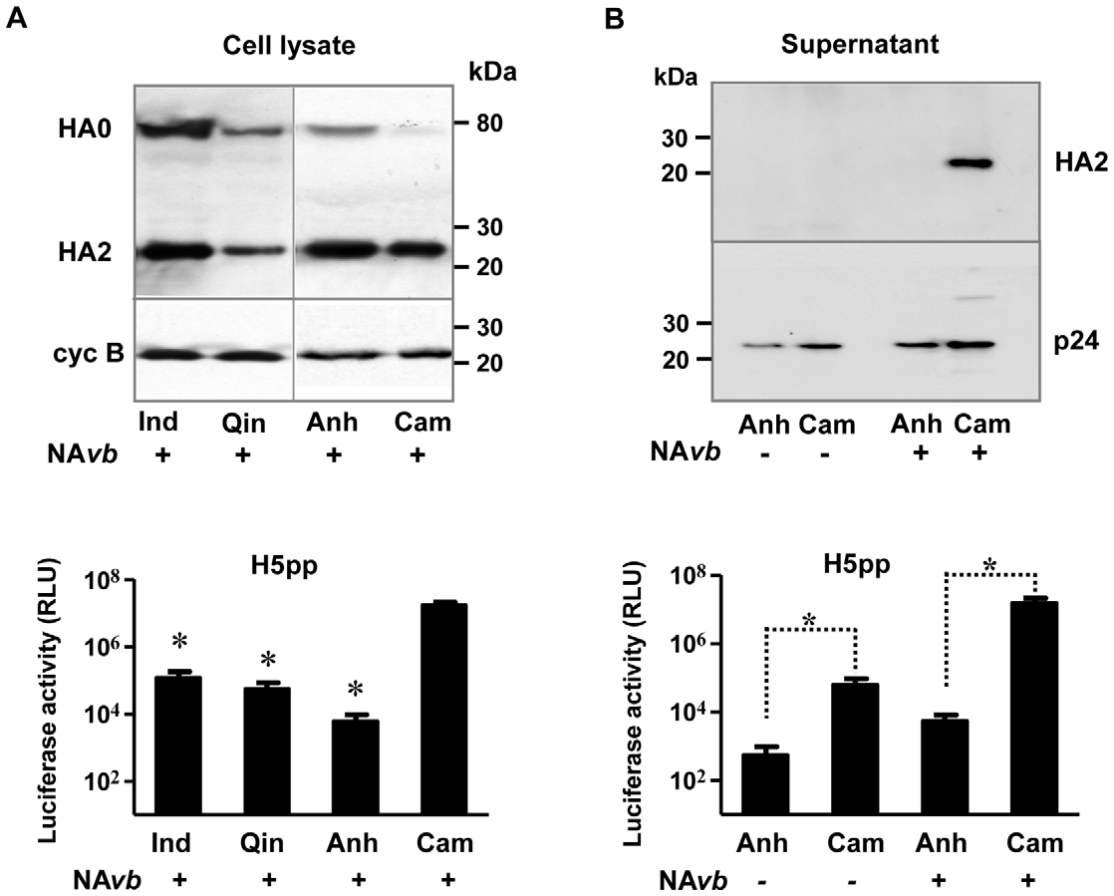
The efficiency of H5pp production does not correlate with protein expression level in producer cells. HEK293T cells were transfected with a lentiviral vector containing gag/pol/luciferase reporter gene, and a plasmid coding for H5-HA from different clades: A/Indonesia/5/2005 (Ind); A/Bar-headed goose/Qinghai/60/2005 (Qin); A/Anhui/1/2005/01 (Anh); A/Cambodia/408008/2005 (Cam). Bacterial neuraminidase from *Vibrio cholerae* (NA*vb*) was added 16 hr post transfection where indicated. Supernatant containing H5pp was harvested at 48 hr and used to transduce MDCK target cells. (A) HA protein expression in cell lysate was analysed using anti-FLAG antibody (upper panel). Cyclophilin B antibody was used as the loading control. Luciferase activity in target MDCK cells was measured 72 hr post transduction (lower panel). Results are shown as means ± SD (n = 4 independent experiments); *p<0.01 compared with H5Cam by the unpaired Student's *t*-test. (B) H5Anh-pp and H5Cam-pp were concentrated by ultracentrifugation. Incorporation of HA into H5pp was determined by western blotting of H5pp pellets using anti-FLAG antibody, as described under Materials and Methods (upper panel). Luciferase activity in MDCK cells was measured 72 hr post transduction (lower panel) and results are shown as means ± SD (n = 4 independent experiments); *p<0.01 by the unpaired Student's *t*-test.

Similar to HA of other subtypes of influenza viruses, H5-HA is highly mutable as a result of antibody-selection pressure, leading to the rise of divergent H5N1 viruses that are categorized into various strains and clades [13], [14], [22]. To ascertain the flexibility and adaptability of H5pp production as an alternative approach for serological studies in the event of novel emerging H5N1 viruses, we sought to develop clade-specific H5pp and compared the ability of three other H5N1 viruses belonging to different clades to pseudotype lentiviral particles. H5-HA from clade 1 (H5Cam), clade 2.1 (H5Ind), clade 2.2 (H5Qin) or clade 2.3 (H5Anh) (see Table 1) was expressed in 293T cells together with lentiviral backbone plasmid to allow the production of H5-pseudotyped lentiviral particles (H5pp). Expression levels of H5-HAs in transfected 293T cells was monitored by Western blot using anti-FLAG antibody directed against the C-terminal tag (Fig. 1A, upper panel). Supernatants containing H5pp were harvested 48 hr post-transfection, and used to transduce MDCK cells for luciferase reporter activity assay (Fig. 1A, lower panel), as described in Materials and Methods. Unexpectedly, we observed significant differences in the transduction of MDCK cells by H5pp, depending on the clades of H5-HAs. In particular, H5Anh from A/Anhui/2005/01 resulted in very low luminescence levels after particle transduction in MDCK cells; whereas H5Cam from A/Cambodia/40808/2005 was the most efficient, inducing a consistent 3–4 log increase in luciferase activity compared with H5Anh (Fig. 1A–B, lower panels). Analysis of cell lysates by Western blots, however, demonstrated that all H5-HAs tested were well expressed in the producer cells and, consequently, that luciferase reporter activity in MDCK target cells did not correlate with the level of HA protein expression in the cells (Fig. 1A). Two main protein bands were detected, consistent with the expected electrophoretic mobility of the uncleaved protein (HA0) and the C-terminal portion of the cleaved form (HA2 subunit), whereas the N-terminal fragment (HA1 subunit) could not be recognized by the anti-FLAG antibody due to C-terminal tagging (Fig. 1A). We next decided to compare in detail the behaviour of H5Cam and H5Anh. To determine whether the difference in luciferase reporter activity was due to the level of H5pp production, culture supernatants containing H5Cam-pp and H5Anh-pp were concentrated by ultracentrifugation, and the resulting H5pp pellets were analyzed by Western blotting. Our results showed that the number of particles produced in the culture supernatant was significantly less for H5Anh than for H5Cam in presence of soluble bacterial neuraminidase, as indicated by lower levels of p24 in concentrated supernatants to detect the lentiviral core and lower luciferase reporter activities in MDCK cells (Fig. 1B). More importantly, incorporation of H5Anh into the pseudotyped lentiviral particles was not observed using anti-FLAG antibody (Fig. 1B, upper panel). Altogether, these data suggest that H5Anh cannot be efficiently incorporated into pseudotyped particles and released into the cell culture supernatant.

**Table 1:** Sequence comparison at the 130-loop region of the receptor binding site of H5 hemagglutinin from different clades and list of mutants tested.

HA	Strain	Clade	130-Loop region (Residue 124–156)*
**Wild type**			
H5Cam	A/Cambodia/408008/2005	**1**	**–** ***S*** **HEAS** ***L*** **GVS** ***AV*** **CPY** ***Q*** **G** ***KS*** **SFFRNVVWLIKKN** ***ST*** **–**
H5Anh	A/Anhui/1/2005/01	**2.3**	**–** ***D*** **HEAS** ***S*** **GVS** ***SA*** **CPY** ***Q*** **G** ***TP*** **SFFRNVVWLIKKN** ***NT*** **–**
H5Qin	A/Bar-headed goose/Qinghai/60/2005	**2.2**	**–** ***D*** **HEAS** ***S*** **GVS** ***SA*** **CPY** ***Q*** **G** ***RS*** **SFFRNVVWLIKKN** ***ST*** **–**
H5Ind	A/Indonesia/5/2005	**2.1**	**–** ***D*** **HEAS** ***S*** **GVS** ***SA*** **CPY** ***L*** **G** ***SP*** **SFFRNVVWLIKKN** ***NA*** **–**
**Mutants**			
AnhM1	A/Anhui/1/2005/01		**–** ***S*** **HEAS** ***L*** **GVS** ***AV*** **CPY** ***Q*** **G** ***KS*** **SFFRNVVWLIKKN** ***ST*** **–**
AnhM2	A/Anhui/1/2005/01		**–** ***S*** **HEAS** ***L*** **GVS** ***AV*** **CPY** ***Q*** **G** ***KS*** **SFFRNVVWLIKKN** ***NA*** **–**
AnhM3	A/Anhui/1/2005/01		**–** ***S*** **HEAS** ***L*** **GVS** ***AV*** **CPY** ***Q*** **G** ***KS*** **SFFRNVVWLIKKN** ***NT*** **–**
AnhM4	A/Anhui/1/2005/01		**–** ***D*** **HEAS** ***L*** **GVS** ***AV*** **CPY** ***Q*** **G** ***TP*** **SFFRNVVWLIKKN** ***NT*** **–**
AnhM5	A/Anhui/1/2005/01		**–** ***D*** **HEAS** ***S*** **GVS** ***AV*** **CPY** ***Q*** **G** ***TP*** **SFFRNVVWLIKKN** ***NT*** **–**
AnhM6	A/Anhui/1/2005/01		**–** ***S*** **HEAS** ***L*** **GVS** ***SA*** **CPY** ***Q*** **G** ***KS*** **SFFRNVVWLIKKN** ***ST*** **–**
AnhM7	A/Anhui/1/2005/01		**–** ***D*** **HEAS** ***S*** **GVS** ***AA*** **CPY** ***Q*** **G** ***TP*** **SFFRNVVWLIKKN** ***NT*** **–**
AnhM8	A/Anhui/1/2005/01		**–** ***D*** **HEAS** ***S*** **GVS** ***SV*** **CPY** ***Q*** **G** ***TP*** **SFFRNVVWLIKKN** ***NT*** **–**
CamM1	A/Cambodia/408008/2005		**–** ***S*** **HEAS** ***L*** **GVS** ***SA*** **CPYQG** ***KS*** **SFFRNVVWLIKKN** ***ST*** **–**
CamM2	A/Cambodia/408008/2005		**–** ***S*** **HEAS** ***L*** **GVS** ***AA*** **CPYQG** ***KS*** **SFFRNVVWLIKKN** ***ST*** **–**
CamM3	A/Cambodia/408008/2005		**–** ***S*** **HEAS** ***L*** **GVS** ***SV*** **CPYQG** ***KS*** **SFFRNVVWLIKKN** ***ST*** **–**

* H5 numbering.

### Swapping of HA2 domain (including the polybasic cleavage site) does not increase production of H5Anh-pp

**Figure 2:**
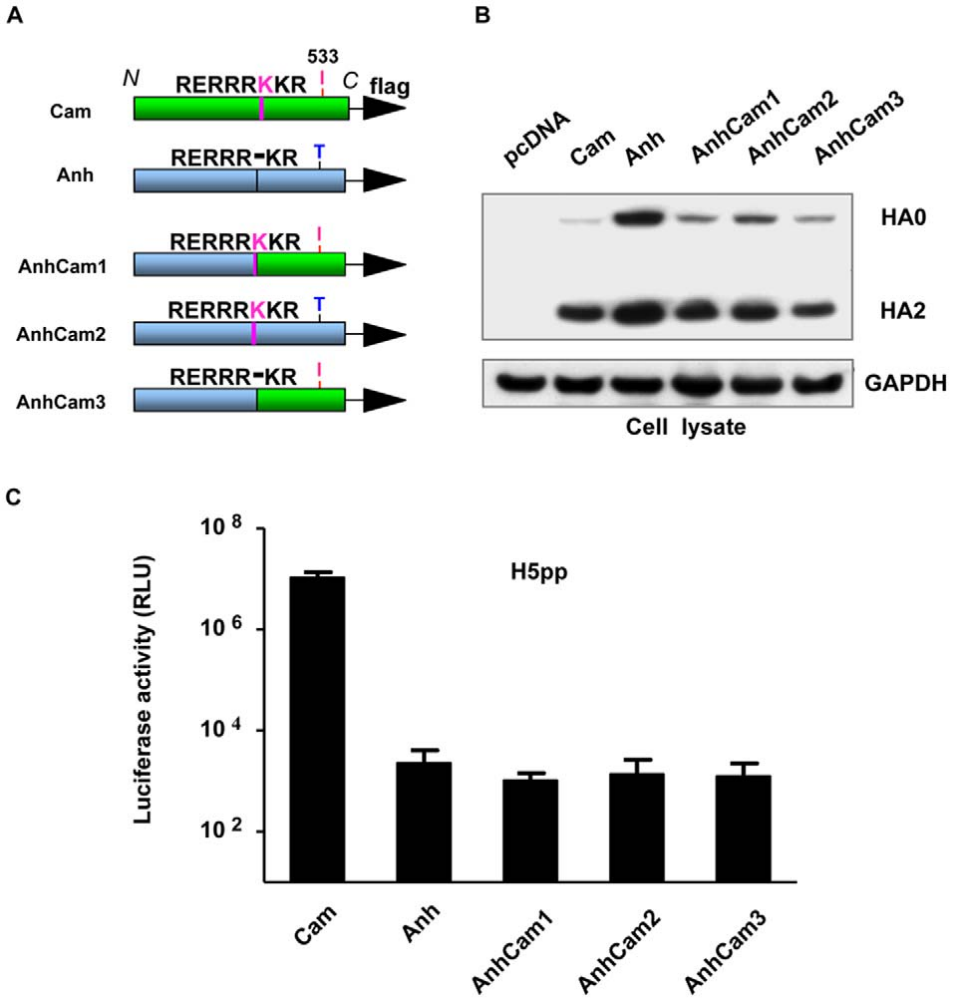
Swapping of HA2 domain (including the polybasic cleavage site) does not increase production of H5Anh-pp. (A) Schematic diagrams of H5Cam (Cam), H5Anh (Anh) and chimerical constructs. (B) HEK293T cells were transfected as described in Fig. 1. NA*vb* was added 16 hr post transfection. At 48 hr post transfection, cell lysates were harvested and analyzed for HA protein expression using anti-FLAG antibody. GAPDH antibody was used as the loading control. (C) MDCK cells were transduced with H5pp containing culture supernatant and luciferase activity was measured at 72 hr post transduction. Results are shown as means ± SD (n = 3 independent experiments). No significant differences were found between Anh and any of the three chimerical constructs AnhCam1, AnhCam2 or AnhCam3.

Sequence analysis of the polybasic cleavage site reveals that H5Anh has a deletion of a lysine residue when compared to H5Cam and moreover, there is an additional amino acid difference in the HA2 region at position 533, which is located at the border between the ecto-domain and the transmembrane domain (TMD) (Fig. 2A). Thus, H5Cam has an isoleucine at position 533 (**I533**), while H5Anh has a threonine (**T533**). Cleavage of HA into HA1 and HA2 subunits by host protease is a critical step for influenza viruses to gain membrane fusion capability [23], [24]; whereas the TMD of HA is important for its association with lipid rafts at the plasma membrane [25]. To test the potential influence of these differences in the cleavage site and at position 533, we generated several chimerical constructs in which either the entire HA2 region including the cleavage site was replaced with that of H5Cam (AnhCam1), or only the cleavage site (AnhCam2) or a single **T533I** amino acid change was introduced (AnhCam3). All constructs were FLAG-tagged at the C-terminal end of H5 sequences as described in the Materials and Methods section (Fig. 2A). When transfected into 293T cells, all three mutant H5Anh proteins were well expressed in the producer cell lysates (Fig. 2B); however, analysis of transduction levels of MDCK target cells by H5pp produced with these H5AnhCam chimerical proteins suggests that none of them was able to increase the production of pseudotyped particles (Fig. 2C). These data indicate that differences in the HA2 domain cannot account for the reduced ability of H5Anh to form pseudotyped particles.

### Two amino acid substitutions in the 130-loop of the receptor binding domain (RBD) are sufficient to confer H5Anh pseudotyping ability

**Figure 3:**
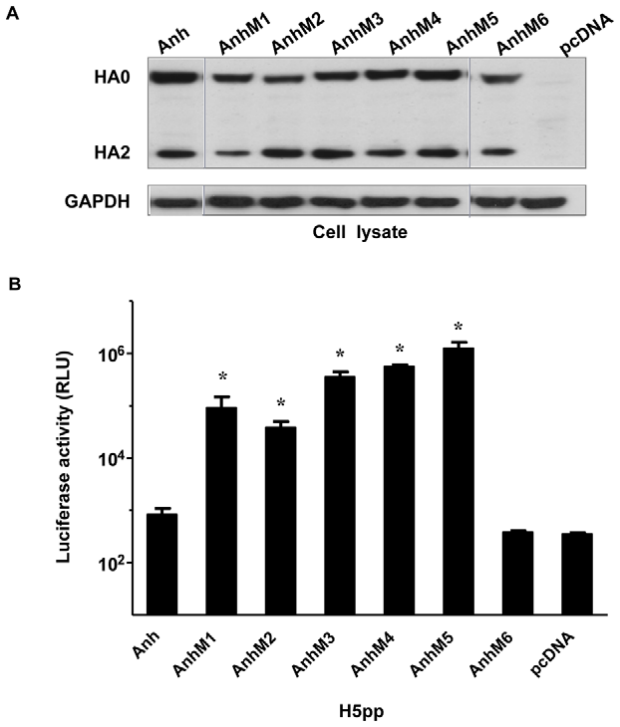
Two amino acid substitutions in the 130-loop of receptor binding domain of H5Anh are sufficient to induce H5Anh-pp production. HEK293T cells were transfected with lentiviral gag/pol with luciferase reporter gene and a plasmid coding for either wild-type or H5Anh mutants described in Table 1. NA*vb* was added 16 hr post transfection. (A) Cell lysates at 48 hr post transfection were analyzed for HA protein expression using anti-FLAG antibody. GAPDH antibody was used as the loading control. (B) Supernatant containing H5pp was harvested and used to transduce MDCK cells. Luciferase activity was measured at 72 hr post transduction and results are shown as means ± SD (n = 3 independent experiments); *p<0.01 compared with H5Anh by the unpaired Student's *t*-test.

Sequence alignment of H5-HA proteins revealed a striking amino acid divergence at 9 positions over a short stretch of only 33-amino-acid-long region around the 130-loop of RBD (Table 1), which accounted for over 30% of the amino acid differences found in the entire HA molecule (576 amino acids in length). Therefore this region was chosen for site-directed mutagenesis to generate a series of H5Anh mutants that were subsequently tested for their ability to pseudotype lentiviral vectors. The level of protein expression for all H5Anh mutants in producer cells was comparable, albeit slightly lower for AnhM1 and AnhM6 (Fig. 3A). Interestingly, all H5Anh mutants that harbored residues alanine-valine at positions 133–134 (AnhM1-5, Table 1) displayed a largely restored ability of H5Anh to produce pseudotyped particles, despite other sequence differences at the 130-loop flanking region (Fig. 3B). By contrast, AnhM6, which contains H5Cam-like 130-loop flanking sequences but serine-alanine at positions 133–134, did not generate efficiently H5Anh-pp in culture supernatant (Fig. 3B). These data clearly demonstrate that amino acid residues at positions 133–134 are crucial for efficient H5pp production. More specifically, substitution of the two amino acids S133-A134 of H5Anh with A133-V134, which are unique to H5Cam sequence, confers H5Anh the ability to be incorporated into the lipid envelope of lentiviral particles and is essential for efficient production of transduction-competent H5Anh-pp.

### A single A134V mutation in the 130-loop of the RBD of H5-HA is the critical determinant for H5pp production

**Figure 4:**
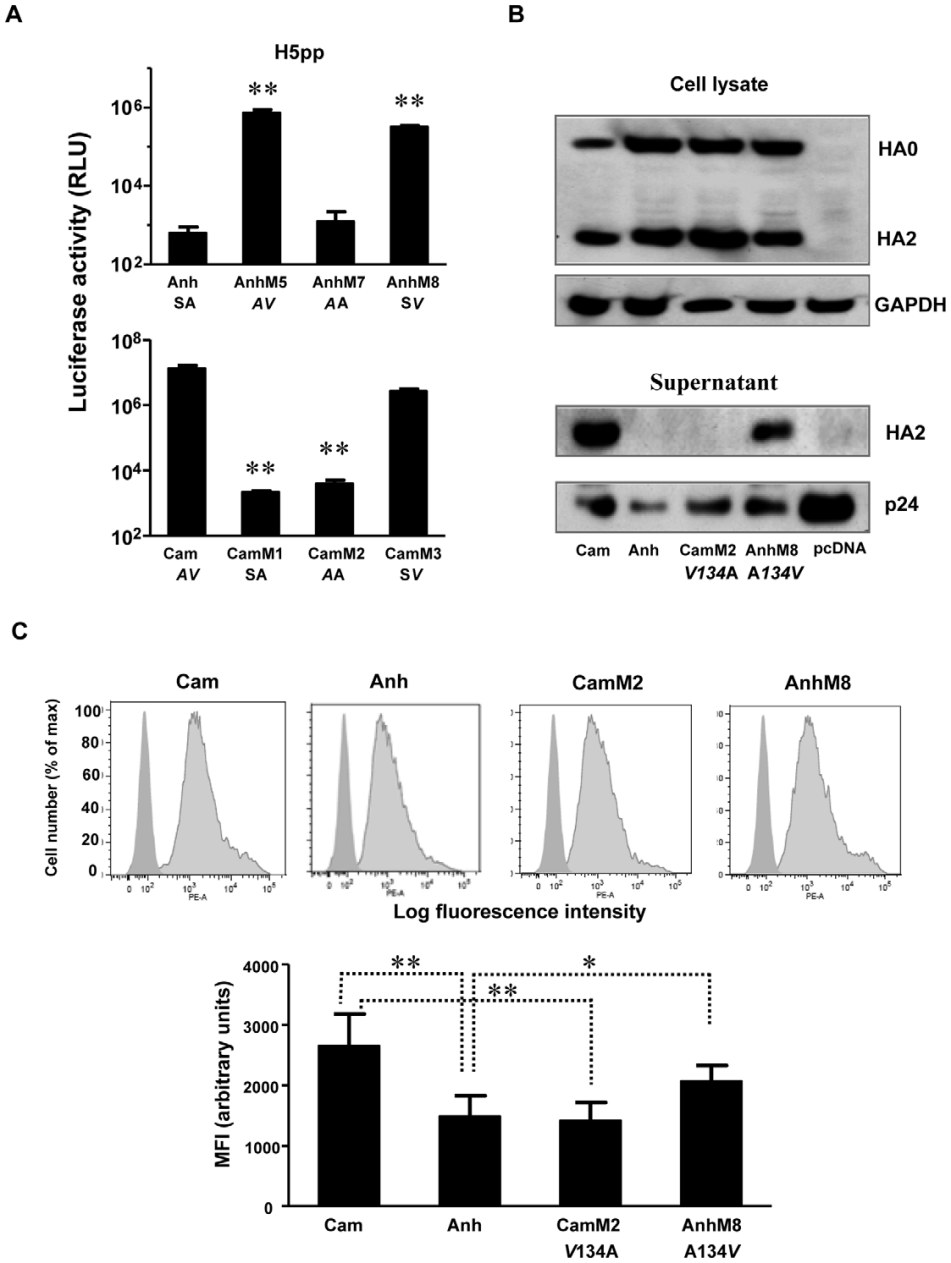
Amino acid residue V134 is a critical determinant for efficient H5pp production. (A) H5pp was produced using H5Anh, H5Cam, their single or double mutants at position 133 and 134. NA*vb* was added 16 hr post transfection. Culture supernatant containing H5pp was harvested at 48 hr post transfection and luciferase activity in MDCK cells was measured at 72 hr post H5pp transduction. Results are shown as means ± SD (n = 3 independent experiments); **p<0.01 compared to their respective wild type HA by the unpaired Student's *t*-test. (B) Cell lysates at 48 hr post transfection were analyzed for HA protein expression using anti-FLAG antibody. GAPDH antibody was used as the loading control. H5pp produced in the supernatant were concentrated by ultracentrifugation and the H5pp pellets were analyzed by western blotting using anti-FLAG and anti-p24 antibodies. (C) HA protein expression at the cell surface was analyzed by immunofluoresent staining followed by flow cytometry as described in Material and Methods. Left and right histograms in the same graph depict cells transfected with pcDNA and H5-HA respectively. Results of mean fluorescence intensity (MFI) are presented as means ± SD (n = 5 independent experiments); **p<0.01; *p<0.02 by the unpaired Student's *t*-test.

To further delineate the respective roles of A133 and V134 for efficient pseudotyping, two additional H5Anh mutants were generated with a single amino acid substitution either at position 133 (**S133A, AnhM7**) or 134 (**A134V, AnhM8**) (Table 1). These experiments revealed that the A134V mutation was sufficient to confer H5Anh the ability to be incorporated into transduction-competent pseudo-particles; whereas the S133A mutation was not (Fig. 4A). We also generated reciprocal mutants of H5Cam that contained either the two residues found at positions 133–134 of H5Anh (viz., **S133-A134**; **CamM1**), or only one single V134A change (**CamM2**), or A133S substitution (**CamM3**) (Table 1). Again, the presence of valine at position 134 was found to be crucial for efficient H5pp production, whereas the A133S substitution had only a marginal effect, consistent with the results obtained with H5Anh mutants (Fig. 4A). To confirm that the effect of valine at position 134 was indeed on the production of H5pp, we analyzed by Western blot both cellular lysates and culture supernatants containing H5pp that were concentrated by ultracentrifugation (Fig. 4B). This series of experiments showed that incorporation of the “Anhui-like” single mutant **CamM2** into the pseudotyped lentiviral particles was below the antibody detection limit, as also seen for wild-type H5Anh; whereas the “Cambodia-like” single mutant **AnhM8** induced H5pp production with an efficiency similar to H5Cam (Fig. 4A–B). Altogether, these experiments demonstrate that valine at position 134 (**V134**) is a critical residue for efficient H5pp production.

### The A134V mutation affects cell surface expression level of HA

Because influenza virus, as well as particles pseudotyped with HA, buds from the plasma membrane [11], [18], we reasoned that changes in surface expression of H5-HA could have an impact on the production of H5pp. Thus, we have compared by flow cytometry plasma membrane expression levels of HA protein in cells transfected with H5Anh and H5Cam. Cells were labelled with an anti-H5N1 antibody, fixed and then stained with a PE-conjugated secondary antibody. As assessed by measuring mean fluorescence intensity (MFI), cell surface expression of H5Anh was significantly less compared to H5Cam (Fig. 4C; p<0.01). Interestingly, introduction of the A134V mutation into H5Anh (AnhM8) increased its cell surface expression (Fig. 4C; p<0.02), and conversely, a V134A mutation in H5Cam (CamM2) reduced transport to the plasma membrane to a level that was not significantly different from that measured with H5Anh (Fig. 4C). These data demonstrate that the Ala to Val substitution at position 134 enhances surface expression of H5-HA.

### The A134V mutation leads to reduced binding to sialic acid receptors

**Figure 5:**
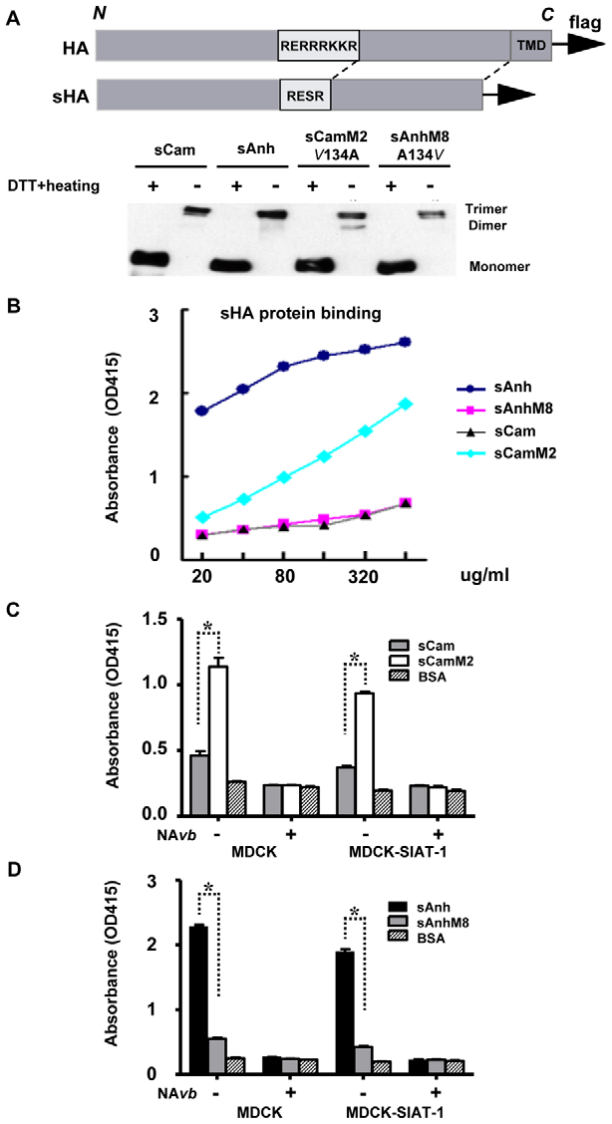
Purification and cell surface binding analysis of recombinant soluble H5-HA (sH5-HA) proteins. (A) Run on a native gel, purified sH5-HA proteins contain mostly the trimeric form. (B) Dose dependent binding of sH5 HA proteins to MDCK cells. Alanine-134 containing HAs (sAnh and sCamM2) bind strongly to MDCK cells, whereas valine-134 containing HAs (sCam and sAnhM8) bind only weakly. Results are plotted as mean values of two independent experiments. (C) Cell surface binding of sAnh and sAnhM8 proteins to MDCK or MDCK-SIAT-1 cells (more alpha-2,6 linked sialic acid than parental MDCK). Cells were seeded in 96-well plate and grown until confluence with or without NA*vb* treatment for 2 hrs prior to fixation in 4% paraformaldehyde. Results are shown as means ± SD (n = 3 independent experiments). (D) Cell surface binding of sCam and sCamM2 to MDCK or MDCK-SIAT-1 cells. Cells were grown and treated as in (C). The results are shown as means ± SD (n = 3 independent experiments). Binding of sH5-HA proteins is dependent of sialic acid at cell surface. Similar results were obtained in MDCK and MDCK-SIAT-1 cells. *p<0.01 by the unpaired Student's *t*-test.

As residue 134 is in the 130-loop of the receptor binding site, we next investigated the impact of A134V mutation on receptor binding properties. We employed a cell-based assay using soluble H5-HA proteins that were engineered by removing TMD and C-tail of HA (Fig. 5A) as described in Material and Methods. Stable cell lines were generated to express sH5Anh, sH5Cam and their reciprocal single amino acid mutant forms (sH5AnhM8 with A134V and sH5CamM2 bearing V134A; see also Table 1), and soluble HA proteins were affinity-purified as described under Material and Methods. When analyzed on native gels, purified soluble H5-HA proteins contained mostly the homotrimeric form (Fig. 5A) that can bind to the sialic acid-containing cellular receptors. We observed that sH5Anh bound strongly to MDCK cells, whereas the A134V mutation reduced the binding to a much lower level (Fig. 5B). By contrast, sH5Cam bound weakly to MDCK cells and, as predicted, the single V134A change induced a major increase in the binding of sH5Cam to MDCK cells (Fig. 5B).

The binding assay was also performed in MDCK-SIAT-1 cells which express two-fold higher amounts of alpha-2,6-link sialic acids than parental MDCK cells [26]. The results obtained were similar to that in parental MDCK cells (Fig. 5C–D). When cells were treated with bacterial neuraminidase NA*vb* before fixation with PFA, the binding of sHA proteins was diminished to background level in both MDCK and MDCK-SIAT-1 cells, indicating that the binding of sHA proteins is sialic acid dependent (Fig. 5C–D).

### Inefficiency of H5Anh-pp production is independent of the lentiviral backbone used

**Figure 6:**
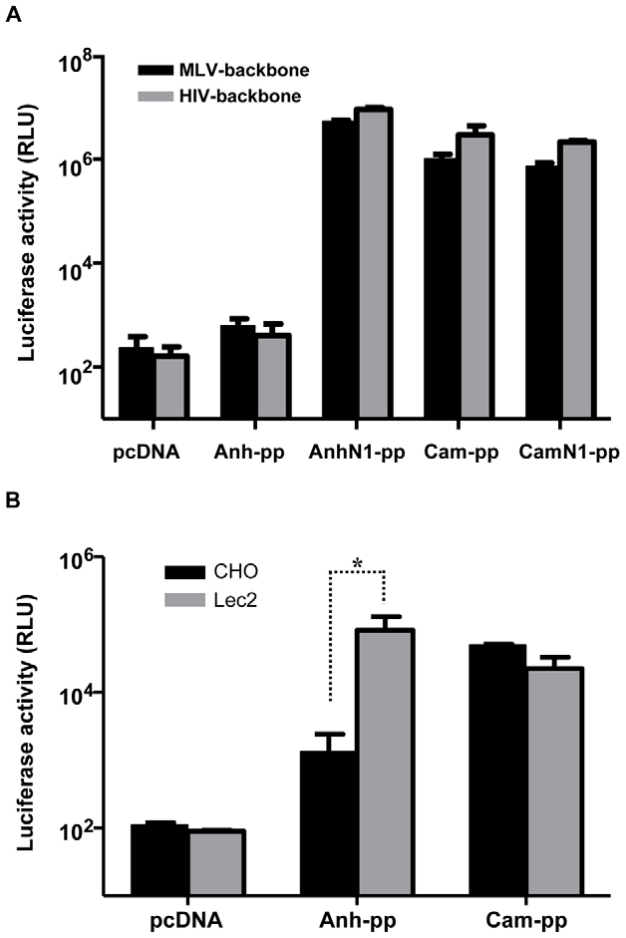
Inefficiency of H5Anh-pp production is independent of the lentiviral backbone used but can be rescued by co-transfection with viral N1 or in sialylation-deficient Lec2 cells. (A) Comparison of H5pp production using HIV and MLV pseudotyping systems. 293T cells were transfected with a plasmid coding for either H5Cam or H5Anh (empty pcDNA vector was used as the negative control, NC), together with either an HIV or MLV lentiviral backbone, as described in Material and Methods. Viral N1 plasmid was included in the transfection mixture where indicated to produce pseudoparticles containing both HA and NA. Culture supernatant containing H5pp was harvested at 48 hr post transfection and luciferase activity in MDCK cells was measured at 72 hr post H5pp transduction. Results are presented as means ± SD (n = 3 independent experiments). No significant differences were found between MLV- and HIV-backbone at all conditions tested. (B) Production of H5Anh-pp is enhanced in sialylation-deficient Lec2 cells. Cells were transfected with HIV gag/pol containing luciferase reporter gene and a plasmid coding for either H5Cam or H5Anh. NA*vb* was added 16 hr post transfection. At 48 hr post transfection, supernatant containing H5pp were harvested from CHO or Lec2 cells and used to transduce MDCK target cells. H5anh-pp production in Lec2 cells was restored to a level similar to that of H5Cam-pp as shown by similar luciferase activity detected in MDCK cells. Results are presented as means ± SD (n = 4 independent experiments); *p<0.01 by the unpaired Student's *t*-test.

Because we had previously found that H5pp with HIV-backbone bud from the plasma membrane in 293T cells [18], a reduced cell surface expression of viral envelope proteins (see Fig. 4C) would be expected to influence the formation of pseudotyped particles and, hence, could account for the observed differences in pseudotyping. It has been reported that retroviruses including HIV and Murine Leukemia Virus (MLV) can also bud from intracellular compartments [27], [28], depending on the cell type and Gag expression systems. Therefore, we also used MLV-based pseudotyping system to compare the efficiency of H5pp production between H5Anh and H5Cam. As demonstrated in Fig. 6A, the results obtained with the MLV-backbone were similar to those with HIV-backbone, thus, indicating that inefficiency of H5Anh-pp production is not a mere consequence of the lentiviral system used for pseudotyping. However, co-transfection of the viral NA from A/Cambodia/JP52a/2005, rescued the inefficiency of H5Anh-pp production (Fig. 6A).

### Production of H5Anh-pp is enhanced in a sialylation-deficient cell line

To further test whether reduced binding to sialic acid receptors, as a result of A134V mutation, is a major contributing factor for pseudotyping efficiency of H5-HA, we examined the production of H5Cam-pp and H5Anh-pp in Lec2 cells which are sialylation-deficient mutants of CHO cells [29]. As H5Cam binds weakly to sialic acid receptors (Fig. 5), NA*vb* added exogenously post transfection was sufficient to release H5Cam-pp into culture supernatant in CHO cells; and the level of H5Cam-pp in CHO cells was not significantly different from that in Lec2 cells (Fig. 6B). Similar to the results obtained in 293T cells, production of H5Anh-pp was lower than H5Cam-pp in CHO cells. By contrast, H5Anh-pp production in Lec2 cells was significantly increased in comparison to that in parental CHO cells and the level of H5Anh-pp obtained in Lec2 cells was similar to that of H5Cam-pp, as indicated by the values of luciferase activity detected in MDCK cells 72 hr post H5pp transduction (Fig. 6B). Together, these findings further suggest that binding of H5-HA to cellular sialic acid containing glycans is a major determinant of H5-HA incorporation into pseudo-particles.

### H5N1 viruses carrying the A134V mutation exhibit reduced capability to agglutinate horse red blood cells

**Figure 7:**
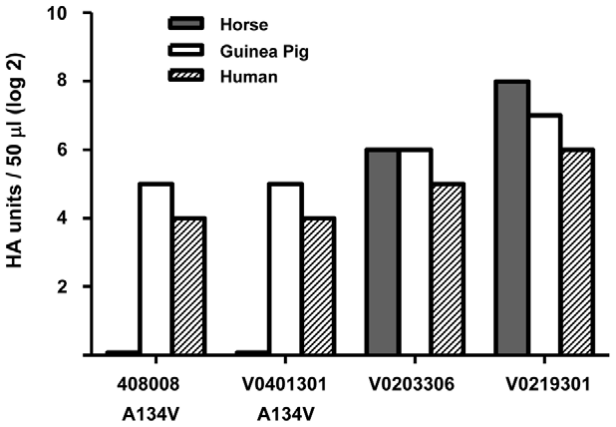
Hemagglutination assay of H5N1 viruses with or without A134V substitution. Comparative hemagglutination ability of four wild-type Cambodian H5N1 isolates: A/Cambodia/408008/2005 (A134V; HA accession number: HQ664938), A/Cambodia/V0401301/2011 (A134V; HA accession number: JN588807), A/Cambodia/V0203306/2011 (A134; HA accession number: JN588805) and A/Cambodia/V0219301/2011(A134; HA accession number: JN588806). Suspensions (0.75%) of human type O, horse and guinea pig blood were used for hemagglutination assays. The two isolates with valine at position 134 were unable to agglutinate horse red blood cells. Results represent the average of two independent experiments.

The reduced binding to sialic acid receptors as a result of the A134V mutation not only leads to changes in pseudotyping efficiency, but is also found to have an impact at the whole virus level. Reverse genetics generated RG-A/Cambodia/408008/2005 with the A134V mutation has been shown to agglutinate to the same degree both human red blood cells (RBCs), which express alpha-2,6-sialic acid, and guinea pig RBCs, which exhibit both alpha-2,3 and alpha2,6-linked sialic acid, but failed to agglutinate horse RBCs, which carry only alpha-2,3-sialic acid [30]. These observations provide an experimental evidence to support the notion that the A134V mutation leads to a reduced alpha-2,3-sialic acid binding of the virus. To confirm the effect of A134V mutation on H5N1 viruses, we performed similar hemagglutination assays using another H5N1 virus strain A/Cambodia/V0401301/2011, which also contains the same A134V mutation. Similar to A/Cambodia/408008/2005, A/Cambodia/V0401301/2011 could agglutinate human and guinea pig RBCs but failed to agglutinate horse RBCs; whereas two other strains of H5N1 viruses without the A134V substitution, isolated in 2011 from human clinical specimens (A/Cambodia/V0203306/2011 and A/Cambodia/V0219301/2011), could also agglutinate horse RBCs (Figure 7).

## Discussion

In previous studies, we have reported the generation of H5pp and have characterized it as a safe alternative to the use of replicative H5N1 virus for sero-surveillance [16], [18]. Because H5pp mimics the entry mechanism of the avian virus while carrying only the H5-HA as envelope protein, it offers the advantage to be specifically neutralized only by anti-hemagglutinin antibodies, avoiding the confounding effect of antibodies directed against N1 neuraminidase due to infection of influenza virus subtypes other than H5N1. We report here that the efficiency to generate HA-only H5pp varies with HAs derived from different H5N1 virus clades, regardless of the lentiviral backbone used. Through serial mutagenesis of two H5-HAs, we have uncovered that differences in receptor binding ability, due to mutations in the receptor-binding domain of HA, may be the underlying mechanism.

It is widely believed that HA is targeted to lipid rafts at the plasma membrane and the transmembrane domain has been described to be important for lipid rafts association of HA [25]. Therefore, we first swapped the transmembrane regions between H5Anh and H5Cam. We also noticed that the cleavage of H5Cam appears to be more efficient (Fig. 1A, 2B). Thus, mutants with or without sequence variations found at the poly-basic cleavage site (AnhCam1, AnhCam2 and AnhCam3) were generated and analysed. However, none of these H5Anh mutants showed appreciable improvement in their ability to generate H5pp, when compared with wild type H5Anh. In fact, the production of H1 and trypsin-dependent H5 pseudo-particles has been reported [31], [32], hence indicating that HA cleavage is not a determining factor for pseudotyping efficiency. Then by multiple sequence alignment, we identified a small region around the 130-loop of the receptor binding site of HA which appeared to be a “hot-spot”, harboring several sequence variations among different H5N1 clades. Through a series of mutagenesis studies, we have found that one single residue at position 134 is a critical switch to dictate the ability of H5 HA to pseudotype lentiviral vectors for the production of H5pp.

Similar to influenza virus, H5pp generated with an HIV-backbone bud at the plasma membrane [18]; therefore the simplest explanation is that the mutation at position 134 may result in a change in cell surface expression of HA. Indeed we have observed a small but consistent change in cell surface HA expression due to mutations at position 134 (Fig. 4C). To exclude the possibility that this finding merely reflected a differential binding to the two HA of the rabbit anti-H5 polyclonal serum (described in Material and Methods), we used another polyclonal serum from a different source (a duck anti-H5 serum described in Ref. 18) and found that the results of cell surface HA staining were similar (data not shown). The fact that the A134V mutation increased cell surface expression of H5Anh, may partially explain the effect of this amino acid substitution on H5pp production. Considering that the variation between H5Cam-pp and H5Anh-pp production resulted in a 3 to 4 log difference in luciferase activity, it is likely that A134V mutation may have an impact on other properties of H5-HA, including binding to sialic acid receptors, which contribute to the observed phenotype. It has been reported in the case of H3-HA pseudotyping that lentiviral particles which incorporate sialic acid binding-incompetent H3-HA (derived from A/Aichi/2/68) can be efficiently generated and released into culture supernatant in the absence of exogenous bacterial NA; whereas the wild-type Aichi-HA fails to do so [33]. Although the difference in pseudotyping observed with wild-type Aichi-HA and its receptor binding-incompetent mutant is diminished when bacterial NA is added, the study by Bosch et al. [33] implies that changes in receptor binding properties can affect pseudotyping efficiency of lentiviral vectors by influenza HA.

Regarding the potential influence of mutations at position 134 of H5-HA on receptor binding properties, there have been reports with contradictory results. First, Yamada et al. [34] found that A134T mutation did not change alpha-2,3 sialic acid binding preference of H5-HA. Then, Auewarakul et al. [35] reported that L129V/A134V allowed for dual binding to both alpha-2,3 and alpha-2,6-sialic acid receptors, although in their study, the effect of A134V mutation alone was not assessed. More recently, using virus elution assay, Imai and colleagues [36] found that H5N1 viruses containing alanine at position 134 (**A134**) show stronger binding than those harbouring threonine (**T134**) to both chicken erythrocytes (expressing both alpha-2,3 and alpha-2,6-sialic acid) and horse erythrocytes (expressing only alpha 2,3-sialic acid). Similar to the observation by Imai et al., we found in the current study that H5Anh which contains A134 displayed a strong binding to both MDCK and MDCK-SIAT-1 cells (expressing an increased level of alpha-2,6 and a decreased level of alpha-2,3-sialic acid than parental MDCK) [26]. As predicted by these observations, the A134V mutation reduced H5Anh binding to a dramatically lower level in both cell lines. It is likely that strong binding of H5Anh to cell surface sialic acid receptors makes it difficult to release H5pp from the producer cells even in the presence of exogenous bacterial NA; and the A134V mutation reduces binding, thus allowing for the release of H5pp. In keeping with this hypothesis, co-transfection with the viral NA gene from H5N1 led to the production of similar amounts of mixed HA-NA pseudoparticles for both H5Anh and H5Cam. Moreover, we did not observe an increase in binding to MDCK-SIAT-1 cells, which contain more alpha-2,6-sialic acids on the cell surface. In fact both sH5Anh and sH5CamM2 bind with slightly lower efficiency to MDCK-SIAT-1, compared with parental MDCK cells, suggesting that A134V mutation probably leads to a decreased binding of H5-HA to alpha-2,3-sialic acid rather than a switch to alpha-2,6-sialic acid binding. Consistent with this notion, we observed an increased level of H5Anh-pp production in Lec2 sialylation-deficient cells, when compared with parental CHO cells (Fig. 6B).

We have found that the A134V mutation not only exerts a critical influence in the determination of pseudotyping efficiency, but has also an impact on H5N1 viruses. Both A/Cambodia/408008/2005 and A/Cambodia/V0401301/2011, two different H5N1 isolates carrying the same A134V mutation could agglutinate human and guinea pig RBCs but failed to agglutinate horse RBCs [30] (also Figure 7 of this paper); whereas two other strains of H5N1 viruses without the A134V mutation could also agglutinate horse RBCs (Figure 7). These observations indicate that A134V mutation in H5-HA reduces virus binding to alpha-2,3-sialic acid. Although co-transfection with viral NA enables efficient lentiviral pseudotyping by H5Anh (Fig. 6A), the differential RBC binding properties observed at the whole virus level, when both HA and NA are present, support the idea that A134V mutation in H5-HA can be biologically relevant. Interestingly, alanine at position 134 (**A134**) is highly conserved in avian H5N1 viruses and so far A134V mutation has only been found in human isolates of H5N1 viruses, both clade 1 and clade 2 viruses isolated from 2004 to 2011. Almost all avian H5N1 isolates possess A134 in the HA. So far only one avian H5N1 virus in the NCBI database has serine instead at position 134 of the HA protein. Notably, more diversity is observed at this position for human isolates of H5N1 viruses: three H5N1 viruses isolated from human patients have a threonine and eleven a valine at position 134 [36]. At least in two cases (A/Cam/408008/2005 and A/Thailand/676/2005), viruses found in the original patient specimens were mixtures of both wild type, containing A134 in the HA, and mutant virus, containing V134 [30], [35]. It is possible that other human isolates of H5N1 viruses may actually contain the A134V mutation but failed to be detected in the process of either virus isolation or traditional capillary sequencing of viral genomes. Thousands of H5-HA sequences are available in the NCBI Influenza Database (http://www.ncbi.nlm.nih.gov/genomes/FLU/FLU.html) from non-human isolates of H5N1 viruses, none of which contains this particular mutation. Altogether, these observations suggest that a valine at residue 134 of the receptor-binding domain is unlikely to be a random sequence variation but may be selected as the avian H5N1 viruses adapt for replication in human hosts. We speculate in general terms that changes in cell surface receptor binding of H5-HA, as a result of A134V mutation, may lead to changes in virus entry and virus release and, therefore, be considered an important factor for determination of host range. It is not clear whether intracellular sialic acid content and distribution may also influence this feature. As our data focus on the pseudotyping system, further studies are required to understand more precisely the biological consequences of A134V mutation and its potential influence on the adaptation of H5N1 viruses in humans.

Our findings have also implications for the applicability of H5pp assay in serological surveys. H5pp has several advantages over the microneutralization method, which is the current gold standard serological assay for the detection of antibodies against avian influenza viruses [37], [38]. Pseudotyped particles are produced from synthetic genes without the need to have access to the virus and can be safely used in BSL-2 containment, making them ideal for widespread use, especially in areas where BSL-3 facilities are not available. Moreover, it has been reported that the H5pp assay is more sensitive than micro-neutralization [16], [39]. It appears, however, that its use in sero-epidemiological studies and to monitor the efficacy of candidate vaccines may be limited by the strain under investigation. Although we and others have found that production of mixed HA-NA pseudo-particles is consistently successful using N1 from either H1N1 or H5N1 [17], [20], [21] (also Fig. 6A of this paper), the production of HA-only pseudotypes would be necessary to eliminate potential cross-reactivity that may be displayed by circulating anti-NA antibodies against N1 from avian H5N1 or seasonal influenza H1N1 [40]. We are cognizant that a single A134V mutation may result in a change of antigenicity but this limitation is not different from that of the microneutralization assay, which is the gold-standard to detect anti-H5 neutralization antibodies and utilizes an available H5N1 virus strain that may not be a perfect match of the viruses associated with the serum samples being tested. In fact, serologic surveys often use a collection of serum samples from human or animals without necessarily knowing the exact virus strain(s) involved. Moreover, if positive, samples shall contain polyclonal antibodies against multiple epitopes to an H5N1 virus, further minimizing the likelihood that a single amino acid substitution would compromise the usefulness of a pseudotype-based serological assay as a safer alternative to the microneutralization test. Careful assessment of the H5pp-based assay should obviously be performed when new strains emerge.

In conclusion, by comparing the ability of different H5-HA to produce pseudotyped particles, we have demonstrated that when a single A134V mutation is introduced in the receptor binding site, the ability of the usually inefficient H5Anh to generate H5pp is largely restored. It is likely that the A134V mutation leads to an increased level of cell surface HA expression and reduced binding to sialic acid receptors, both of which contribute to the production of H5pp. The A134V mutation has been reported as a naturally occurring mutation in human host; and importantly, this mutation is so far only found in human isolates of H5N1 viruses. Our data with hemagglutination assays further demonstrate that viral isolates from human cases with avian influenza carrying the A134V substitution exhibit a reduced binding to alpha-2,3 linked sialic acids. Therefore, our results may have implications for the understanding of human host adaptation of avian influenza H5N1 viruses. It is possible that other mutations leading to reduction in receptor binding may exist and cause a change in pseudotyping efficiency. Thus, H5pp production together with soluble HA protein cell binding analysis may serve as convenient functional assays to screen for mutations with potential consequences on receptor binding properties and host adaptations of H5N1 viruses. Although zoonotic transmission from poultry to humans remains inefficient for H5N1, it may be of importance to monitor closely mutations in regions of the receptor binding site of H5-HA.

## Materials and Methods

### Cells

293T, MDCK, CHO and Lec2 cell lines were obtained from ATCC (Manassas, VA, USA). MDCK-SIAT-1 cells were generated by stable transfection of human alpha-2,6-sialyltransferase in MDCK cells and was described elsewhere [26]. This cell line over-expresses alpha-2,6-linked sialic acid compared to parental MDCK [26]. 293T, MDCK and CHO cells were cultured at 37°C with 5% CO2 in Dulbecco's Modified Eagle's Medium (DMEM, Invitrogen, Carlsbad, CA, USA) supplemented with 10% fetal bovine serum (FBS, Invitrogen) and 1% penicillin-streptomycin. MDCK-SIAT-1 cells were grown in DMEM containing 10% FBS and 1 mg/ml G418. Lec2 cells, which lack terminal sialic acid in their glycoproteins and gangliosides due to a defect in the CMP-sialic acid transporter [29], were cultured in Minimum Essential Medium (MEM-alpha, Invitrogen) supplemented with 10% FBS and 40 ug/ml L-proline.

### Plasmids

H5Cam (HQ664938, see also ref. 30), H5Anh (ABD28180), H5Ind (ABP51969), H5Qin (ABE68923)), mutants AnhM1-M6, CamM1-M3 (Table 1) and N1 gene (ABO10176) from A/Cambodia/JP52a/2005 were synthesized as human codon optimized genes (GENEART®, Regansburg, Germany) and subcloned into the mammalian expression vector pcDNA3.1 (Invitrogen). Mutants AnhM7 and AnhM8 were generated by site-directed mutagenesis using QuikChange site-directed Mutagenesis Kit (Stratagene, Santa Clara, CA, USA) according to the manufacturer's instructions. To generate soluble H5-HA constructs, the transmembrane domain (TMD) of the HA was removed, and the polybasic cleavage site was changed into a monobasic cleavage site RESR by site-directed mutagenesis to avoid the potential influence of H5-HA cleavage in cells on the purification of sHA proteins, which involve multiple steps. The truncated HAs were then subcloned into pcDNA3.1 (Invitrogen). All H5 plasmids were tagged with the FLAG-epitope at the C-terminal and sequenced to confirm that they contain only the expected mutations as indicated in Table 1.

### Production of H5pp

The production of lentiviral particles pseudotyped with H5 hemagglutinin was performed as previously described [18]. Briefly, HEK293T cells were co-transfected with a plasmid containing the coding sequence of the indicated H5-HA and a lentiviral backbone plasmid pNL-Luc E− R− which carries a modified proviral genome of HIV with *env* deleted and is engineered to express the firefly luciferase reporter. Alternatively, MLV-backbone plasmids (a kind gift from Dr. Michael Farzan, Harvard Medical School)described in [41] were used where indicated. To release particles into the culture medium, either soluble bacterial NA from *Vibrio cholerae* (NA*vb*; Roche, Mannheim, Germany) was added to the producer cells at a concentration of 6.25 mU/ml or co-transfection of N1 gene was used where indicated. Supernatants containing H5pp were harvested 48 hr post-transfection, filtered and used to transduce MDCK cells for luciferase reporter activity assay or concentrated by ultracentrifugation as indicated.

### Luciferase reporter activity assay

MDCK cells (4000 cells/well) were seeded in 96-well white assay plates one day before H5pp transduction. Luciferase reporter activity assay was performed 72 hr post transduction using Bright-Glow Luciferase substrate (Promega, Mandison, WI, USA) according to the manufacturer's instructions. Samples were measured using a Microbeta Luminometer (PerkinElmer, Waltham, MA, USA) and data were expressed as Relative Luminescence Units.

### Western blots

Equal amounts of protein from total cell lysates or equal volumes of H5pp concentrated by ultracentrifugation were subjected to sodium dodecyl sulfate-polyacrylamide gel electrophoresis (SDS-PAGE). Proteins were then transferred onto Hybond-P polyvinylidene difluoride (PVDF) membranes (Invitrogen) that were blocked with 5% milk for 30 min at room temperature. H5-HA was detected by incubation for 2 hr at room temperature with a mouse monoclonal anti-FLAG M2 antibody (Sigma, St.Louis, MO, USA; 1∶1000 dilution) conjugated with horse radish peroxidase (HRP) (Sigma). The core protein in the pseudotyped particles was detected using an anti-p24 antibody (Abcam, Cambridge, UK) for 1 hr at room temperature at a 1∶1000 dilution, followed by an additional 1 hr incubation with a goat-anti-mouse secondary antibody conjugated with HRP (Zymed®, Invitrogen) at a 1∶5000 dilution. The levels of cyclophilin B (detected with a rabbit anti-cyclophilin B antibody from Abcam, 1∶5000 dilution) or GAPDH (detected with a mouse anti-GAPDH antibody from Abcam, 1∶10000 dilution) were measured on the same blots to verify that equal amount of samples had been transferred. Proteins were visualized by chemiluminescence using ECL Western blot detection reagents (Invitrogen). The relative electrophoretic mobility was estimated using Novex® Sharp Pre-stained Protein Standards (Invitrogen).

### Surface labelling of H5-HA

293T cells transfected with H5 HA were detached with and resuspended in PBS, blocked in 10% horse serum and then labelled with a polyclonal rabbit anti-H5N1 antibody (Sino Biologicals Inc., Beijing, China) at a 1∶400 dilution for 1 hr at 4°C. Unbound antibodies were removed by washing three times with cold PBS, followed by staining with a phycoerythrin (PE)-conjugated, donkey-anti-goat secondary antibody (Jackson Immunoresearch Laboratories, Suffolk, UK) for 30 min at 4°C. Data were collected from at least 5000 cells on an LSRII flow cytometer (BD Biosciences, Franklin Lakes, NJ, USA) and post-acquisition analyses of cell surface expression of H5-HA was performed using FlowJo software (TreeStar, Ashland, OR, USA).

### Expression and purification of soluble H5-HA proteins

HEK293T cells stably expressing soluble HA (sHA) proteins were generated by selection of transfected cells in culture medium containing 300 ug/ml hygromycin for at least 1 month. To purify sHA proteins, cells were grown in DMEM with 5% FBS until 90% confluence. Culture supernatant containing secreted sHA proteins was cleared by centrifugation at 4000 rpm for 15 minutes at 4°C, concentrated by Amicon Ultra-15 Centrifugal Filter Units with Ultracel-100 membrane (Millipore, Billerica, MA, USA; 100 kDa cut-off) and stored at −80°C until use. Soluble HA proteins were affinity purified from concentrated supernatant using anti-FLAG M2 affinity gel (Sigma). Because it has been shown that HA-bound sialic acid could interfere with the accessibility of the receptor-binding site to cellular receptors [42], anti-FLAG M2 resins bound with sHA proteins were washed twice in cold PBS and subjected to treatment with NA*vb* (Roche, 62.5 mU/ml) at 37°C for 45 minutes to remove terminal sialic acid residues, followed by three washes in cold PBS. Bound sHA proteins were eluted with 150 µl FLAG peptide (International Laboratory, USA, 0.4 mg/ml in PBS) for four times. To remove the FLAG peptides, all eluates were pooled and concentrated using Amicon Ultra-0.5 mL with Ultracel-100 membrane (Millipore, 100 kDa cut-off). To examine the oligomeric state of sHAs, proteins were resolved on a discontinuous native PAGE (6% of acrylamide) followed by western blot detection using a HRP-conjugated anti-FLAG M2 antibody (Sigma).

### Cell-based HA binding assay

MDCK cells were grown in 96-well plate until complete confluence, then fixed with 4% paraformaldehyde (PFA, Sigma), washed three times in PBS and blocked for at least 2 hrs in 5% BSA. The indicated amount of purified soluble HA proteins, measured by the Bradford assay, were added to the wells in duplicates or triplicates and incubated overnight at 4°C. Cells were washed three times in PBS and then incubated with anti-FLAG antibody (Origene, Rockville, MD, USA; 1∶1000 dilution for 2 hrs at room temperature) to detect sHA proteins bound to cell surface. After washing for three more times in PBS to remove unbound sHA proteins, cells were incubated with goat-anti-mouse secondary antibody conjugated with HRP (Zymed®, Invitrogen) at a 1∶5000 dilution for 1 hr at room temperature. Unbound secondary antibody was removed by washing three times in PBS, and ABTS substrate (Invitrogen) was added to the plate according to the manufacturer's instructions. Forty minutes after the addition of substrate, absorbance at 415 nm (OD415) was measured using a Sunrise™ plate reader (Tecan, Männedorf, Switzerland).

### Virus isolation

Cambodian H5N1 virus strains were isolated from human clinical specimens by inoculation in Madin-Darby canine kidney (MDCK) cells in the biosafety level 3 laboratory of the Institut Pasteur in Cambodia, according to conventional protocols [43].

### Hemagglutination assay

Hemagglutination titres were measured using 0.75% suspensions of human (type O), horse and guinea pig red blood cells, as previously described [30], [43].

### Statistical analysis

Results are presented as mean values ± SD of the indicate number of observations. Statistical difference between groups was determined by the unpaired Students's t-test with a 0.05 significance level.
